# Examining boys’ and girls’ health‐related quality of life from the first to the third year of upper secondary school: A prospective longitudinal study

**DOI:** 10.1002/nop2.366

**Published:** 2019-08-21

**Authors:** Ingrid Oma Langeland, Ragnhild Sollesnes, Roy Miodini Nilsen, Grethe Almenning, Eva Langeland

**Affiliations:** ^1^ Department of Health and Caring Science, Faculty of Health and Social Sciences Western Norway University of Applied Sciences Bergen Norway; ^2^ Department of Health and Functioning, Faculty of Health and Social Sciences Western Norway University of Applied Sciences Bergen Norway; ^3^ Section for Innovation, Health, Children and Youth, Department of Health and Care Bergen Municipality Bergen Norway

**Keywords:** adolescents, nurses, nursing, public health nursing, quality of life

## Abstract

**Aim:**

To examine differences in health‐related quality of life between boys and girls in the first and third years of upper secondary school.

**Design:**

Prospective longitudinal study.

**Methods:**

The KIDSCREEN‐10 was used to assess health‐related quality of life. Differences in health‐related quality of life over time were estimated using a linear mixed‐effects model for correlated measurements.

**Results:**

In the first‐year boys (*N* = 168) and girls (*N* = 228) reported a mean health‐related quality of life score of 76.3 (*SD* 10.7) and 69.8 (*SD* 11.5), respectively. In the third year, the mean health‐related quality of life score for boys and girls was 73.5 (*SD* 12.4) and 65.7 (*SD* 13.3), respectively. Boys had a significant decrease in health‐related quality of life mean score of −2.6 and girls a significant decrease of −3.8 (*p* < .001) over the 3‐year period. There was no significant difference between boys’ and girls’ health‐related quality of life changes (*p* = .39).

## 1 INTRODUCTION

Health‐related quality of life (HRQoL) can be viewed as a psychological construct that describes the physical, mental, social, psychological and functional aspects of well‐being and function from a personal perspective (Ravens‐Sieberer & Bullinger, 1998). The World Health Organization points out that adolescent health and well‐being are essential for healthier and more sustainable societies (WHO, 2017b). In recent years, HRQoL in adolescents has received more attention from researchers in clinical practice and governments (Ravens‐Sieberer et al., 2014; Wallander & Koot, 2016; WHO, 2015). There is a stronger focus on understanding, mapping and improving adolescents’ health, well‐being and quality of life (Deighton et al., 2014; Solans et al., 2008; WHO, 2014). Measuring adolescents’ HRQoL should be based on self‐reports, especially in populations that seem to be healthy (Rajmil, López, López‐Aguilà, & Alonso, 2013; Wallander & Koot, 2016). Instruments can provide important information about adolescents’ health status and help to identify populations at risk (Ravens‐Sieberer et al., 2014). Furthermore, subjective health and perceived well‐being are considered important aspects of promoting health to adolescents (Gaspar et al., 2009) and finding strategies to promote health among adolescents in school is vital (Svedberg, Eriksson, & Boman, 2013). Haraldstad, Christophersen, Eide, Natvig, and Helseth (2011b) point out that knowledge about predictors of HRQoL is of particular interest for public health nurses. Furthermore, events and difficulties that emerge late in students’ schooling careers are often not detected (Dupéré et al., 2015). Gillison, Skevington, and Standage (2008) point out that identifying at‐risk groups and individuals for whom intervention may be particularly crucial is an important area for future research. Differences in HRQoL and health across countries and even in nation states show the importance of national contexts for adolescent well‐being, HRQoL and health (Michel, Bisegger, Fuhr, & Abel, 2009; Patton et al., 2016). There is rather limited research on adolescents’ HRQoL over time through upper secondary school in the Norwegian context.

## 2 BACKGROUND

Adolescents generally have good health and HRQoL, but there remain health challenges in the adolescent population (Patton et al., 2016; WHO, 2014, 2017a). Previous studies have indicated gender differences and found that girls tend to report lower HRQoL than boys (Hourani, Hammad, Shaheen, & Amre, 2016; Limperg et al., 2014; Meyer, Oberhoffer, Hock, Giegerich, & Müller, 2016; Ravens‐Sieberer et al., 2014). Studies have also found that self‐reported mental health problems are related to decrease HRQoL (Otto et al., 2017; Sharpe et al., 2016). Research has revealed that adolescents’ HRQoL decreases with age (Bolton et al., 2014; Meade & Dowswell, 2015) and that girls’ HRQoL declines more than boys’ with increasing age (Michel et al., 2009).

A longitudinal study concludes that changes over time are gender‐related (Meade & Dowswell, 2016). In that study, males reported significantly higher HRQoL than females across three of five dimensions of the KIDSCREEN‐27 instrument: physical and psychological well‐being, autonomy and parent relations (Meade & Dowswell, 2016). A follow‐up study conducted by González‐Carrasco, Casas, Malo, Viñas, and Dinisman (2017) indicates a reduction in subjective well‐being with increasing age, and this reduction is more pronounced in girls. Palacio‐Vieira et al. (2008) found during a three‐year follow‐up period, in the whole sample, a small to moderate decline in HRQoL. When the selection was stratified by gender and age, the reduction in HRQoL was particularly noticeable in the 13–17 age group, while in the 18–21 age group there was a more stable development of HRQoL. Improvement was also found in some of the dimensions in a study by Palacio‐Vieira et al. (2008). Rajmil et al. (2009) found that children and adolescents reported lower HRQoL after three years, but the decline was sharper for those with mental health problems. Otto et al. (2017) also found that an increase in mental health problems was associated with reductions in children's and adolescents’ HRQoL over time and improvement in protective factors such as self‐efficacy and social support was positively associated with improvement in HRQoL. The study by Gillison et al. (2008) indicates that quality of life of adolescents is stable over a one‐year period without health threats.

As shown above, relatively a few longitudinal studies have been performed on adolescents’ HRQoL. Accordingly, the main purpose of the present study is to examine whether there are any differences in HRQoL among boys and girls in the first and third years of upper secondary school and any differences in the degree of change during these 3 years.

Based on previous research, we hypothesized the following points:

1. Girls report lower HRQoL than boys in the first and third years in upper secondary school.
2. For both girls and boys, HRQoL decreases from the first to the third year of upper secondary school and the change is significantly different between girls and boys.

## 3 METHODS

### 3.1 Design

This is a longitudinal study with two repeated measures of the individuals. The first measurement was carried out in the first year of upper secondary school during 2014–2015, and the second measurement of the same boys and girls was conducted in the period January–March 2017. The study was reported according to STROBE guidelines (Supporting Information).

### 3.2 Sample and procedure

The schools were selected from a large city in western Norway. In this city, many upper secondary schools measure the pupils’ HRQoL as a mandatory routine in the first year. The schools were included in the study if they fulfilled the following inclusion criteria: the students had completed the KIDSCREEN‐10 index in the first year of upper secondary school (2014–2015 school year) and the schools had specialized education programmes in general studies and supplementary general studies. A total of 11 schools met the criteria. Written requests were sent to the principals and the school nurses. Five schools wanted to be part of the study. The five schools were from four different parts of the city, and both private and public schools took part. The students were included if their KIDSCREEN‐10 index from the first year of upper secondary school was available and saved in health records by the school health services. Students who received information about the study and gave written consent for their KIDSCREEN‐10 index data to be collected from the medical journal and agreed to fill out a new KIDSCREEN‐10 index in the third year of upper secondary school participated.

**Figure 1:**
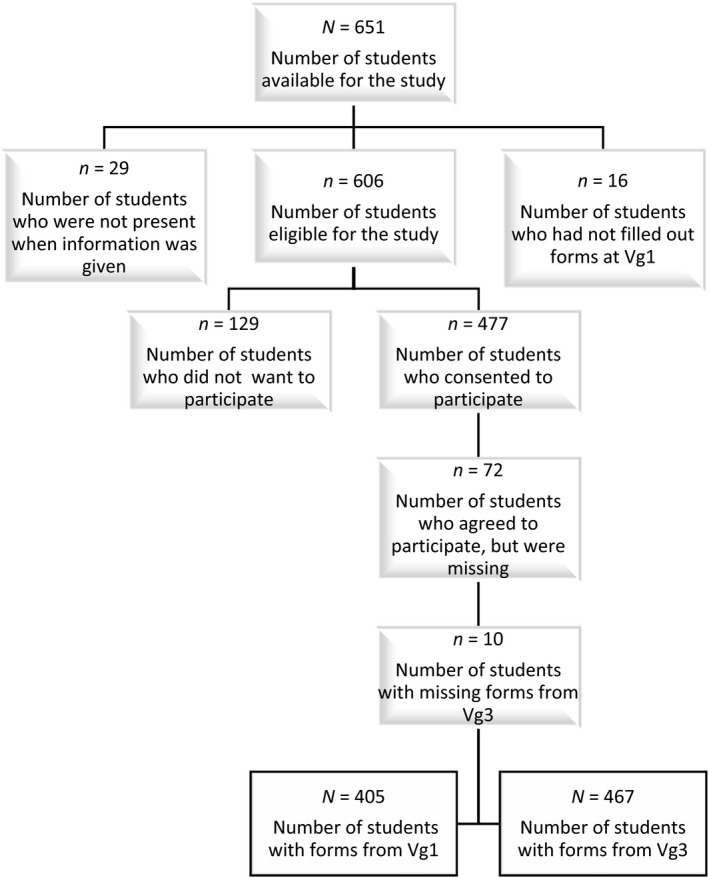
Flow chart of the sample in the study

A total of 651 students from 22 classes were suitable for the study. Twenty‐nine students were not present when the information was given, and 16 students had not completed the KIDSCREEN‐10 index in the first year of upper secondary school. Moreover, 129 students did not want to participate, while 477 returned written consent forms. There were 72 students whose KIDSCREEN‐10 indexes from the first year were missing from the medical records. Ten students did not fill out a new KIDSCREEN‐10 index in the third year. There were 405 students with data from the first year: 228 girls and 168 boys, with nine who did not report their gender in the initial index. However, in the third year there were data from 467 students: 270 girls and 197 boys. See the flow chart in Figure 1.

### 3.3 Ethical considerations

The study was conducted according to the World Medical Association’s (2013) rules and principles to ensure good ethical medical and health research. All adolescents in the study were over 16 years old in the third year and gave written informed consent before participating in the study. The study was approved by the Regional Committees for Medical and Health Research Ethics (2016/1257/REC North).

Written requests were sent to the municipality and the county municipality, and information was sent to all schools. To ensure that the information was as similar as possible throughout the sample, the information was read aloud in all classes. The students were informed that participation was voluntary and that they could withdraw without further explanation. All students received time to consider whether to participate. After written consent was obtained, the students took 10–15 min to complete the KIDSCREEN‐10 index on paper in the classroom. The first author was always present in the classroom or at school to answer questions. The school nurse was available when the study was completed.

### 3.4 Data collection

#### 3.4.1 Sociodemographic variables

The following sociodemographic variables were collected in the first year, in addition to the responses in the instrument: gender, age, education programme, disease, residential status and siblings.

#### 3.4.2 Health‐related quality of life

We used the KIDSCREEN‐10 index generic questionnaire to map adolescents’ HRQoL at both measurement times. Three versions of the KIDSCREEN questionnaires were available for children/adolescents or parents (the proxy version). The KIDSCREEN instruments are designed to measure self‐reported HRQoL in healthy and chronically ill children and adolescents from 8 to 18 years of age and to identify individuals at risk in terms of their subjective health (Ravens‐Sieberer et al., 2006). The instrument was developed through a collaboration between 13 European countries (Ravens‐Sieberer et al., 2006). The KIDSCREEN instruments were translated into Norwegian according to international standards (Haraldstad, Christophersen, Eide, Natvig, & Helseth, 2011a). Reliability was measured, with a Cronbach's *α* of .81, which corresponds to that reported by Ravens‐Sieberer et al. (2010). In the present study, Cronbach's *α* was .77 for the first year and .79 for the third year. Therefore, the reliability of the KIDSCREEN‐10 index is considered to be satisfactory according to Connelly (2011).

The KIDSCREEN‐10 index includes physical, psychological and social components, and the 10 questions with Likert scales for responses yield an overall HRQoL score (Ravens‐Sieberer et al., 2006). The answers are based on the previous week. Unlike other HRQoL instruments, the KIDSCREEN‐10 index measures both positive and negative aspects of life (Wallander & Koot, 2016). Eight questions are formulated positively, while questions three and four are formulated negatively. These two questions must be reverse scored, as higher values always indicate higher HRQoL. Scores can be transformed to a 0–100 scale, with 100 being the top score. In line with cutoff scores used in clinical practice and European norms for the KIDSCREEN‐10 index, students with scores of over 65 were considered to have good to high HRQoL and students with a score of 65 or less were seen as having low HRQoL.

Self‐assessed health was measured as a single question scored 1–5, where 1 indicates poor and 5 indicates excellent health. This question is not included in the overall HRQoL score. This score was converted to a 0–4 scale to compare the results of the study with those of other representative studies in Norway.

### 3.5 Statistical analysis

The present study examined differences in HRQoL between boys and girls in the first and third years of upper secondary school and changes in HRQoL for boys and girls from the first to the third year. For this purpose, we used a linear mixed‐effects model that allows for the correlation of individual measurements over time and allows some individual measurements to be missing, provided that their absence is random. Our model defined gender, time and the gender‐by‐time interaction as fixed effects, whereas a random intercept was specified to account for correlated measurements of the same individual over time. We present estimates as mean differences with 95% confidence intervals and p values. To examine further whether changes in HRQoL were different for boys and girls over time, we compared the likelihood between models with and without the gender‐by‐time interaction using the likelihood ratio test. The analyses were performed with and without adjustment for age and residential status. We did not adjust for other covariates, which did not vary significantly between boys and girls. We used a multivariate normal multiple‐imputation method (200 imputation sets) to replace missing data for age (*N* = 10) and residential status (*N* = 173).All statistical analyses were performed using Stata SE software version 15(StataCorp).

## 4 RESULTS

The sample in the first year consisted of more girls *N* = 228, 57.6%) than boys (*N* = 168, 42.4%). Most of the adolescents were 15–16 years old when the first measurement was carried out. Most of those in the sample in this study were students attending education programmes for specialization in general studies. In the first year of upper secondary school, 75.8% of the girls reported no disease. Similarly, 77.9% of the boys reported no disease in the first year. Furthermore, 68.4% of the girls and 82.4% of the boys reported that they lived with both parents. In addition, 68.7% of the girls and boys reported living with their siblings in the initial measurement. Sample characteristics for the girls and boys in the first year of upper secondary school are shown in Table 1.

**Table 1:** Sample characteristics for girls and boys in the first year of upper secondary school

Characteristic at VG1	Gender		*p* value^a^
	Girls, *N* (%)	Boys, *N* (%)	
Total	228 (57.6)	168 (42.4)	
Age			
15/16	224 (98.2)	157 (94.0)	.025
>16	4 (1.8)	10 (6.0)	
Educational programme			
Specialization	206 (90.4)	150 (89.3)	.728
Vocational	22 (9.6)	18 (10.7)	
Disease			
No	169 (75.8)	127 (77.9)	.625
Yes	54 (24.2)	36 (22.1)	
Residential status			
Mother and father	117 (68.4)	103 (82.4)	.007
Others^b^	54 (31.6)	22 (17.6)	
Siblings			
Yes	119 (70.0)	85 (66.9)	.572
No	51 (30.0)	42 (33.1)	

a By chi‐square test.

b Mother, father, grandparents, stepparents, girlfriend and boyfriend.

Girls had lower mean HRQoL scores than boys in both the first and third years. In the first year, the mean HRQoL score for boys and girls was 76.3 (*SD* 10.7) and 69.8 (*SD* 11.5), respectively (Tables 2 and 3; *p* < .001). In the third year, the mean HRQoL scores for boys and girls were 73.5 (*SD* 12.4) and 65.7 (*SD* 13.3), respectively (Table 3; *p* < .001). Boys in this sample had a decrease in HRQoL mean of − 2.6 (*p* < .001) and the girls a decrease of − 3.8 (*p* < .001) over a three‐year period (Table 3). However, there was no significant difference in change in mean HRQoL scores from the first to the third year between boys and girls (*p* = .39).

**Table 2:** Mean for overall HRQoL score (0–100 scale) at the first measurement point (Vg1)

Characteristic at VG1	KIDscreen (HRQoL)			Self‐assessed health		
	No.	Mean (*SD*)	*p* value^a^	No.	Mean (*SD*)	*p* value^a^
Total	405			404		
Gender						
Girls	228	69.8 (11.5)	<.001	228	2.67 (0.9)	<.001
Boys	168	76.3 (10.7)		167	3.20 (0.9)	
Age						
15/16	381	72.6 (11.6)	.883	381	2.91 (1.0)	.025
>16	14	72.1 (11.4)		13	2.31 (0.9)	
Educational programme						
Specialization	361	73.0 (11.2)	.093	361	2.94 (0.9)	.005
Vocational	44	69.2 (14.5)		43	2.51 (1.1)	
Disease						
No	304	73.8 (10.8)	.001	303	3.05 (0.9)	<.001
Yes	91	69.1 (13.0)		91	2.42 (1.1)	
Residential status						
Mother and father	225	73.8 (10.8)	.020	224	3.02 (1.0)	.003
Others^b^	79	70.3 (13.2)		79	2.65 (0.9)	
Siblings						
Yes	208	73.3 (10.7)	.566	207	2.99 (0.9)	.083
No	97	72.4 (13.3)		97	2.77 (1.0)	

Abbreviation: HRQoL, health‐related quality of life.

a By Independent *t* test.

b Mother, father, grandparents, stepparents, girlfriend and boyfriend.

**Table 3:** Mean and difference in overall HRQoL outcome (0–100 scale), the first and second measurement time

HRQoL outcome	Gender				Mean gender difference	
	Girl		Boy			
	No.	Mean (*SD*)	No.	Mean (*SD*)	Crude (95% CI)^a^	Adjusted (95% CI)^b^
KIDscreen						
Vg1 Baseline	228	69.8 (11.5)	168	76.3 (10.7)	6.5 (3.78, 9.24)*	6.1 (3.69, 8.50)*
Vg3 Follow‐up	270	65.7 (13.3)	197	73.5 (12.4)	7.7 (5.18, 10.30)*	7.2 (4.82, 9.64)*
Change (95% CI)^a^		−3.8 (−5.49, −2.19)*		−2.6 (−4.55, −0.70)*	0.35^c^	0.39^c^
Self‐assessed health						
Vg1 Baseline	228	2.67 (0.93)	167	3.20 (0.92)	0.52 (0.30, 0.73)*	0.52 (0.34, 0.71)*
Vg3 Follow‐up	268	2.62 (0.96)	196	3.05 (0.96)	0.43 (0.23, 0.63)*	0.45 (0.27, 0.64)*
Change (95% CI)^a^		−0.03 (−0.16, 0.09)		−0.12 (−0.26, 0.31)	0.41^c^	0.48^c^

Abbreviations: CI, confidence interval; HRQoL, health‐related quality of life; *SD* standard deviation.

a From a linear mixed‐effects model with a random intercept.

b Adjusted for age and residential status; missing data for age (*N* = 10) and residential status (*N* = 173) was imputed using a multiple‐imputation technique.

c *P* for interaction was obtained using a likelihood ratio test for time‐by‐gender interaction, that is to test whether change in outcome from Vg1 to Vg3 was different for boys and girls.

* *p* < .001.

In the present study, we found that 29.4% of the students rated their HRQoL poorly (HRQoL score ≤ 65.00) in the first year and 40% of the students rated their HRQoL poorly (HRQoL score ≤ 65.00) in the third year.

We also used self‐assessed health as a marker for HRQoL. Similar to the results of KIDSCREEN, girls had lower mean scores in self‐assessed health than boys in both the first and third years (Table 3). However, there was no significant change in self‐assessed health from the first to the third year for either boys or girls (Table 3). Furthermore, there was no significant difference in the changes in mean self‐assessed score from the first to the third year between boys and girls (*p* = .48).

In total, 8.7% of the students reported low self‐assessed health (scores of 0–1) in the first year of upper secondary school. A total of 10.8% students reported low self‐assessed health in the third year. Spearman's rho showed a moderate positive correlation between HRQoL and self‐assessed health in the first year (0.628) and third year (0.617).

## 5 DISCUSSION

Hypothesis (1) that girls would report lower HRQoL than boys in the first and third years of upper secondary school was supported. Girls reported a significantly lower HRQoL than boys at both measurement points. This finding is consistent with those of previous studies of gender differences between girls and boys, with girls reporting lower HRQoL than boys (Hourani et al., 2016; Limperg et al., 2014; Meyer et al., 2016; Ravens‐Sieberer et al., 2014).

Hypothesis (2) was that HRQoL would decrease from the first to the third year of upper secondary school for both girls and boys and that the change would be significantly different between the genders. Hypothesis (2) was only partially confirmed. In the present study, we found a significant decrease in both girls’ and boys’ HRQoL from the first to the third year. This finding is in line with previous longitudinal research indicating a decline in adolescents’ HRQoL and well‐being over time (Palacio‐Vieira et al., 2008; Rajmil et al., 2009). The finding also adds to previous research that has shown a more pronounced decrease in girls’ HRQoL than boys’ over time (González‐Carrasco et al., 2017; Meade & Dowswell, 2016). However, it was not confirmed that the decrease in girls’ HRQoL was significantly different from that of boys. It was difficult to find a basis of comparison for longitudinal research on differences between the genders in the change from the first to the third year. Consequently, there is a need for more research.

The other independent variables, such as age, education programme, disease, residential status and siblings in our study could not explain the decline in HRQoL over time, either in girls or boys. In comparison, Otto et al. (2017) found that an increase in mental health problems was associated with decreasing HRQoL over time and that improvements in self‐efficacy and social support were both associated with improved HRQoL over time. The cross‐sectional study by Haraldstad et al. (2011b) found that the difference in HRQoL between boys and girls disappeared when they controlled for other variables such as pain and negative body image. Previous studies have also shown that bullying is associated with lower HRQoL and increased subjective health problems (Carlerby, Viitasara, Knutsson, & Gadin, 2013; Haraldstad et al., 2011b; Menrath et al., 2015). Other factors related to adolescents’ quality of life and HRQoL that have been detected are socioeconomic status (Baumann, Chau, Kabuth, & Chau, 2014; Scott et al., 2016), sleep problems (Gustafsson et al., 2016; Svedberg et al., 2013) and being overweight (Jalali‐Farahani, Chin, Amiri, & Mohd Taib, 2014). Wilson and Cleary (1995) point out this complexity in measuring HRQoL, meaning that it is important to model possible factors that may affect the outcome. Therefore, like Otto et al. (2017), we acknowledge that it would provide important insights to include more clinical variables in this study. However, we were dependent on clinical variables that had been previously obtained in connection with the KIDSCREEN‐10 index in the first year, limiting our ability to add more clinical variables in the initial state. This shows that we need more research on factors that might predict development in adolescents’ HRQoL.

In our study, we detected a moderate positive correlation between HRQoL and self‐assessed health at both measurement points. In line with Jerdén, Burell, Stenlund, Weinehall, and Bergström (2011), our study indicates that girls’ self‐assessed health is significantly lower than that of boys. A total of 91.3% of the students in the first year and 89.2% of the students in the third year rated their health as good. Our study does not explain why more girls than boys rated their health poorly, or why many students reported that their health was good despite poor HRQoL scores. However, previous research has shown that girls, in particular, can experience strong inner demands that are perceived as stressful and that both girls and boys rated the demands of school as stressful (Wiklund, Malmgren‐Olsson, Öhman, Bergström, & Fjellman Wiklund, 2012). Our finding corresponds with those of other studies that although adolescents may experience subjective health complaints and stress, they still report good health (Breidablik, Meland, & Lydersen, 2008; Wiklund et al., 2012).

There was no significant change in girls’ or boys’ self‐assessed health from the first to the third year of upper secondary school. This finding is in line with that of Vie, Hufthammer, Holmen, Meland, and Breidablik (2014), who also found self‐rated health to be relatively stable over time.

In this present study, we found that 29.4% of the students in the first year and 40% in the third year rated their HRQoL poorly. This finding may indicate an additional sensitive period in upper secondary school. Therefore, the measurement and improvement of both girls’ and boys’ HRQoL should be of special interest for schools and their health services (Dupéré et al., 2015; Haraldstad et al., 2011b). Patton et al. (2016) highlight that countries that already have good participation in upper secondary school in a worldwide context should seek to further improve the health and well‐being of adolescents, and they revealed evidence that a positive school ethos was associated with health. The World Health Organization (WHO, 2017b) calls for prevention, early detection and treatment of problems in the adolescent population, so it is crucial that the school health services, and the schools cooperate to promote and implement measures to enhance students’ HRQoL throughout upper secondary school.

### 5.1 Limitations and strengths

This study has some limitations. A challenge in a longitudinal study is a loss of participants both before (response bias) and through the study period (attrition bias) (Polit & Beck, 2017). The participants in this study were collected from schools where the students had completed the KIDSCREEN‐10 index in the first year of upper secondary school as part of the health services’ mandatory routine. Therefore, a strength of this study was the opportunity first to obtain indexes from the students in the third year and then collect their KIDSCREEN‐10 indexes from the first year. A reversed data collection method reduced the risk of dropout from the study. In comparison with other longitudinal studies, a response rate of 65.3% in the first year and a response rate of 77.1% in the third year could be considered as acceptable (Meade & Dowswell, 2016; Palacio‐Vieira et al., 2008).

Self‐selection into the study could lead to biases resulting in pre‐existing differences between groups and could threaten internal validity (Polit & Beck, 2017). The schools in this study represent different districts in the city. Furthermore, there were both vocational schools and schools with specialized programmes in general studies in the sample, and both public and private schools were included. Therefore, the schools in this study may represent the upper secondary schools in Norway.

Another limitation is that we lack information about students who did not take part in the study. Previous research has detected that non‐respondents generally tend to have poorer health (Bandayrel & Johnston, 2014). Delfabbro, Winefield, Winefield, Malvaso, and Plueckhahn (2017) found that the students who dropped out of the study were not very different to those who participated in relation to variables of interest such as psychological well‐being, self‐esteem and suicidality. For this reason, it is uncertain whether the findings in this study would be different with a higher response rate.

Validity depends on ongoing evidence building (Polit & Beck, 2017). An important question in this context is whether the KIDSCREEN‐10 index is sensitive enough to measure change in adolescents’ HRQoL over time and give valid results (Polit & Beck, 2017; Solans et al., 2008). The review article by Deighton et al. (2014) indicated that there was too little evidence that the KIDSCREEN instruments could be routinely used to capture change over time. However, they could not exclude the possibility. The study by Palacio‐Vieira et al. (2010) indicates that the KIDSCREEN‐52 instrument seems sensitive enough to detect possible changes over a three‐year period. Moreover, Ravens‐Sieberer et al. (2010) point out that the short version KIDSCREEN‐10 index has many of the same characteristics and benefits as the other KIDSCREEN instruments.

## 6 CONCLUSION

This study indicates that, in a sample of Norwegian adolescents, there is a gender difference in HRQoL throughout upper secondary school. Girls reported significantly lower HRQoL than boys in the first and third years. However, this study also revealed a significant decline in HRQoL for girls and boys over this period. The decline may indicate that these three years may be a particularly sensitive period for both genders. In line with previous research, this study also highlights the need for longitudinal studies that provide insight into adolescents’ HRQoL using more clinical variables (Otto et al., 2017; Wilson & Cleary, 1995). Moreover, there is need for longitudinal research that investigates differences in the extent of change between the genders from the first to the third year of upper secondary school.

Our study also indicates that the short version KIDSCREEN‐10 index may be used to capture change in adolescents’ HRQoL over a three‐year period. However, further research is needed to gather more evidence.

### 6.1 Relevance to clinical practice

Although adolescents generally have good health and the HRQoL of youths has attracted more attention in clinical practice in recent years, this study indicates a potential to sustain and improve adolescents’ HRQoL throughout upper secondary school in a Norwegian context. This may indicate a need for a broader approach and for school health services and schools to collaborate on interventions to strengthen both genders’ HRQoL throughout upper secondary school.

## CONFLICT OF INTEREST

The authors declare that they have no conflict of interest.
